# Age at onset of epilepsy shapes neurocognitive profiles in focal cortical dysplasia

**DOI:** 10.1007/s00415-025-13090-4

**Published:** 2025-05-03

**Authors:** Anna-Laura Potthoff, Lukas Tennie, Juri-Alexander Witt, Attila Rácz, Valeri Borger, Hartmut Vatter, Albert Becker, Rainer Surges, Matthias Schneider, Christoph Helmstaedter

**Affiliations:** 1 Department of Neurosurgery, University Hospital Bonn, Venusberg Campus 1, 53127 Bonn, Germany; 2 Department of Epileptology, University Hospital Bonn, Venusberg Campus 1, 53127 Bonn, Germany; 3 Department of Neuropathology, University Hospital Bonn, Venusberg Campus 1, 53127 Bonn, Germany

**Keywords:** Focal cortical dysplasia, Epilepsy, Age at onset of epilepsy, Neurocognition, Intelligence quotient

## Abstract

**Background:**

Focal cortical dysplasia (FCD) is a common developmental brain disorder frequently associated with refractory epilepsy and neurocognitive comorbidities. This study examines the neurocognitive profiles of patients with FCD, with particular attention to histopathological classification, age at onset of epilepsy (AOE), FCD lateralization and localization, and antiseizure medication (ASM) load.

**Methods:**

This study was conducted on 98 patients with FCD (type IIa: n = 26, type IIb: n = 59) who had undergone surgical treatment for epilepsy. Patients underwent comprehensive presurgical neuropsychological assessments for intelligence (IQ) and six cognitive domains.

**Results:**

Patients with FCD type IIb significantly more often exhibited an earlier AOE (< 6 years, 65.5% vs. 38.5%, *p* = 0.021) and a longer duration of epilepsy at the time of cognitive testing (mean ± SD, 18.8 ± 13.61 vs. 11.88 ± 9.09 years, *p* = 0.008) compared to patients with FCD type IIa. The most notable differences in cognitive performance were observed between patients with early (< 6 years) and late AOE (≥ 6 years) for IQ and motor functions. In these domains, patients with early AOE consistently scored lower (IQ: 2.24 ± 1.17 vs. 2.79 ± 0.83, *p* = 0.021, impaired patients: 36% vs.15.8%; motor function: 1.46 ± 1.05 vs. 2.25 ± 0.95, *p* = 0.002, impaired patients: 74.4% vs. 43.8%). Differences in cognitive performance were not linked to FCD type, lateralization, localization, or ASM load.

**Conclusion:**

Our findings indicate that the AOE emerged as the determining factor for cognitive performance in refractory epilepsy patients due to FCD. As expected in cases of early childhood onset epilepsies, a neurodevelopmental disruption particularly of IQ and motor function was seen.

**Supplementary Information:**

The online version contains supplementary material available at 10.1007/s00415-025-13090-4.

## Introduction

Focal cortical dysplasia (FCD) belongs to the heterogeneous class of malformations of cortical development, originating during embryonic and fetal brain development [1, 2]. FCD commonly presents with symptoms of epilepsy, with frontal and temporal lobe epilepsy being frequently reported [3, 4]. Despite treatment with antiseizure medications (ASMs), a significant percentage of patients, estimated between 30 and 70%, continue to suffer from intractable seizures [5–7]. In addition to epilepsy, cognitive impairments are prevalent in individuals with FCD [8, 9].

Studies consistently show below-average performances across various cognitive domains and developmental delays [10–13]. Although some research suggests that neurocognitive effects are more severe in patients with FCD type I [12, 14, 15], other studies find greater cognitive impairments in those with type II [16, 17]. Notably, there have been limited investigations comparing the neuropsychological profiles of the different histopathological FCD types and their influence on cognition remains inconclusive [9, 16].

In addition, there is evidence suggesting that the age at onset of epilepsy (AOE) is a factor that must be considered in relation to neurocognitive outcomes [18, 19] given that early seizures and interictal epileptic discharges can have a negative impact on cognitive development.

This study aims to determine if different types of FCD, here mostly FCD type IIa and FCD IIb, which vary in clinical characteristics and histopathology, exhibit distinct presurgical cognitive profiles. Besides the histopathological types, factors such as AOE, dysplasia localization (frontal vs. temporal vs. posterior) and lateralization (right vs. left), ASM drug load (number of concomitant ASM), and seizure frequencies were examined as potential mediator variables. Consequently, presurgical neurocognitive test data from nearly 100 patients diagnosed with FCD, assessed across seven neuropsychological domains, were analyzed.

## Methods

### Study design

This study retrospectively analyzed patients with pharmacoresistant epilepsy who underwent surgery at the University Hospital Bonn between 1989 and 2017, meeting the inclusion criteria of histopathologically confirmed FCD and available comprehensive presurgical neurocognitive data. Patient specific clinical information included histopathological FCD type, localization and lateralization of the FCD, AOE, duration of epilepsy, presurgical seizure frequency, and ASM drug load were assessed. MRI characteristics were not analyzed due to the absence of standardized imaging data across the entire cohort. Based on the theoretical implications of neuronal plasticity until the age of six, as suggested by Helmstaedter et al. [20], all patients were divided into two groups: patients with an early onset of epilepsy (AOE < 6 years) and patients with a late onset of epilepsy (AOE ≥ 6 years). Seizure frequency was categorized according to the classification proposed by Vickrey et al. [21]. As all patients experienced more than 10 seizures per year, they were assigned to one of three subgroups: a mild to moderate group (fewer than one seizure per week), a severe group (one or more seizures per week but less than one per day), and a very severe group (one or more seizures per day).

### Epilepsy diagnostics and presurgical evaluation

All patients underwent comprehensive presurgical epilepsy diagnostics at the Department of Epileptology, University Hospital Bonn. The diagnostic procedure followed a specific algorithm designed to identify individual characteristics of patient’s epilepsy [22] and mandatorily include video-electroencephalography (EEG) recordings and magnetic resonance imaging (MRI). In selected cases further assessment was necessary.

### Neuropsychological assessment

A comprehensive neuropsychological assessment was conducted as part of the presurgical evaluation [23]. The assessment covered seven cognitive domains, including intelligence (in terms of intelligence quotient (IQ) as a global estimate of cognitive abilities), motor functions, attention, memory (verbal and figural), language, and visuo-construction. The tests were administered and evaluated by a neuropsychologist or trained assistant at the Department of Epileptology, University Hospital Bonn (for detailed information, see Supplementary Table S2). Since patients of varying ages were evaluated, in part different test instruments were used. Consequently, all test results were standardized into scores according to the norms of each respective test. The scores for each cognitive domain were then summarized into a categorical scale ranging from 0 to 4 [24, 25]. Categories represented different levels of performance: 0 (“far below average”), 1 (“below average”), 2 (“low average”), 3 (“average”), and 4 (“above average”) (Table 1). The assignment of a category for each cognitive domain was carried out according to the following scheme: If all test scores fell within one SD range of the mean of the respective norms, the category assigned was 3 (“average”). Categories 4 (“above average”) were assigned if at least two test scores were above one SD or one test score was above two SDs. Category 2 (“low average”) was assigned in cases where one test score lay below one SD. Conversely, categories 1 (“below average”) were assigned if at least two scores were below one SD or one test score fell below two SDs, and category 0 (“far below average”) if two or more test scores were below two SDs. The difference between two adjacent categories reflects about one SD when calculating the mean standard value of all test scores of the respective domain and indicated significantly better or poorer performance [26]. Complete test data across all cognitive domains were not available for all patients. However, no systematic association was found between missing data and lesion lateralization or date of surgery.

**Table 1:** Performance categories

Category	Label	Description
0	Far below average	Two or more test scores below − 2 SD from the norm
1	Below average	At least two test scores below − 1 SD, or one below − 2 SD
2	Low average	One test score below − 1 SD
3	Average	All test scores within ± 1 SD of the normative mean
4	Above average	At least two test scores above + 1 SD, or one score above + 2 SD

### Neuropathological examination

The processing of resected brain tissue samples was conducted at the Department of Neuropathology, University Hospital Bonn. The classification system according to Blümcke et al. was employed to distinguish between FCD types [27].

### Statistics

The statistical analyses were based on the categories from each cognitive domain, minimizing missing values per patient and cognitive domain. This approach allowed for calculating a domain category even when individual tests were absent or different test instruments were used due to age differences.

For statistical analyses, the categories were treated as interval-scaled data which allowed the calculation of parametric tests. Means (M) were calculated as a measure of central tendency and standard deviations (SDs) were calculated as a measure of dispersion. In addition, percentages were provided to demonstrate individual cognitive impairments. Univariate ANCOVAs, using the preoperative number of ASMs as a covariate, were conducted separately for each cognitive domain and factor. Performing separate ANCOVAs for each domain helped mitigate the impact of missing values, as not all patients were assessed in every cognitive domain. At the individual level, the frequencies of impaired individuals in each cognitive domain were analyzed and compared between factor levels. The “impaired” category encompassed all patients whose cognitive scores were rated below average, including those classified as “low average”. Frequency distributions were analysed using χ^2^ tests. The significance level was set to 0.05. Statistical results were rounded to the second decimal place. All statistical analyses were conducted using IBM SPSS Statistics for Macintosh, version 23 (IBM Corp., Armonk, N.Y., USA).

Plotting was performed using GraphPad PRISM (Version 10.2.3) and R (Version 4.4.1) using the packages ‘networkD3’, ‘dplyr’, ‘ggplot2’, and ‘htmlwidgets’. The assembly of the plots was completed with Adobe Illustrator (Version 28.1).

## Results

### Patient demographics and clinical characteristics

**Fig. 1:**
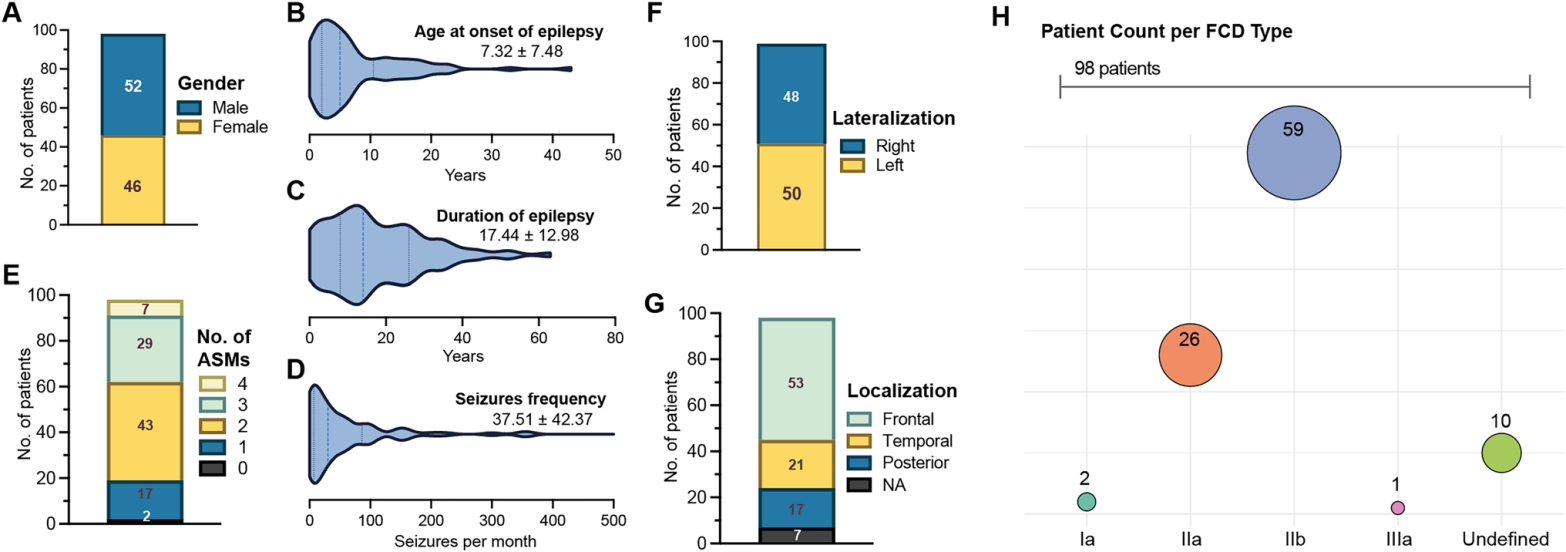
Patient demographics and characteristics. **A** Stacked bar plot depicting the number of female and male patients. **B**–**D** Violin plots illustrating the distribution of age at onset of epilepsy, duration of epilepsy, and seizure frequency. Mean ± SD are indicated in each plot. **D** Two patients with very high seizure frequency (1,200 and 4,500 seizures per months) were excluded from the plot as outliers. **E** Stacked bar plot showing the number of ASMs at time of cognitive assessment. **F**–**G** Stacked bar plots depicting the number of patients according to lateralization (**F**) and localization (**G**). **H** Bubble plot displaying the number of patients across different histopathological types. *ASMs* antiseizure medication, *FCD* focal cortical dysplasia, *NA* not applicable, *No.* number

The study cohort comprised a total of 98 patients who had undergone presurgical neurocognitive testing (Fig. 1, Supplementary Table S1). Among these patients, 52 (53%) were male and 46 (47%) female (Fig. 1A). The mean AOE was 7.32 ± 7.48 years (range: 0–43) (Fig. 1B), and the mean duration from the onset of epilepsy to presurgical neurocognitive testing was 17.44 ± 12.98 years (range: 0–63) (Fig. 1C).

The average number of seizures per month among these patients was 37.51 ± 42.37 (range: 0–150) (Fig. 1D). At time of cognitive assessment, 81% of the patients were treated with ASM polytherapy, defined as taking two or more ASMs (Fig. 1E).

Lateralization was evenly distributed between the right (49%) and left (51%) hemispheres (Fig. 1F). Most FCDs were located in the frontal lobe (54%), followed by the temporal lobe (22%) and posterior regions (17%) (Fig. 1G). Among the histopathological subtypes, type II was predominant, accounting for 87% of cases (Fig. 1H).

Epilepsy more frequently started before the age of 6 (early AOE) and was predominantly located extratemporally (frontal: 55%, temporal: 22%, posterior: 16%) in patients with FCD type IIb.

**Fig. 2:**
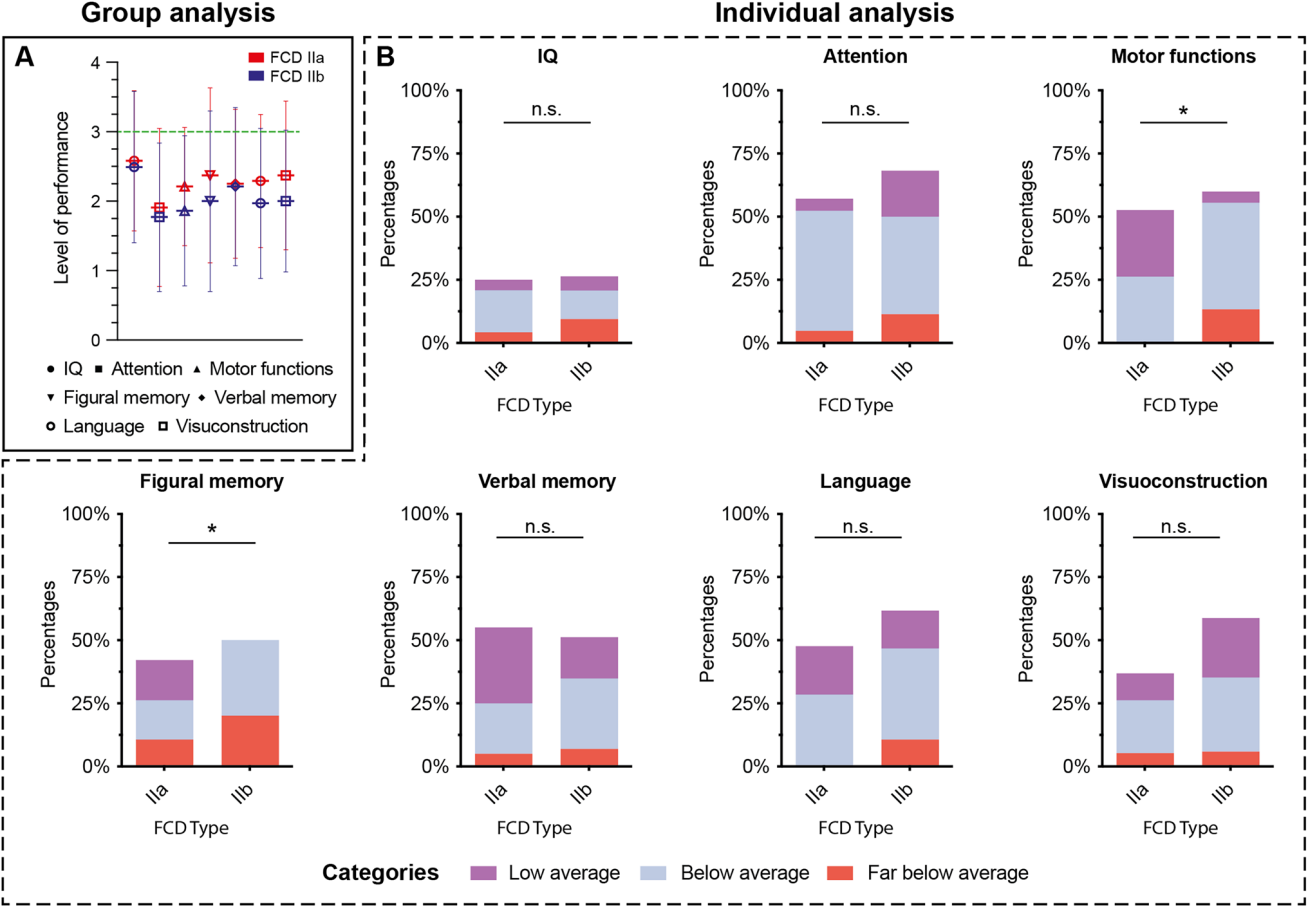
Cognitive performance across cognitive domains in patients with FCD types IIa versus IIb. **A** Group analysis results: Average cognitive performance scores for patients with FCD Type IIa and IIb, presented as mean ± SD. The green line indicated the performance level ‘average’. **B** Individual analysis results: Proportion of impaired patients categorized as borderline, below average, and far below average based on their performance in neurocognitive assessments. *FCD* focal cortical dysplasia, *IQ* intelligence quotient, *n.s.* not significant, *SD* standard deviation, * *p* < 0.05

**Fig. 3:**
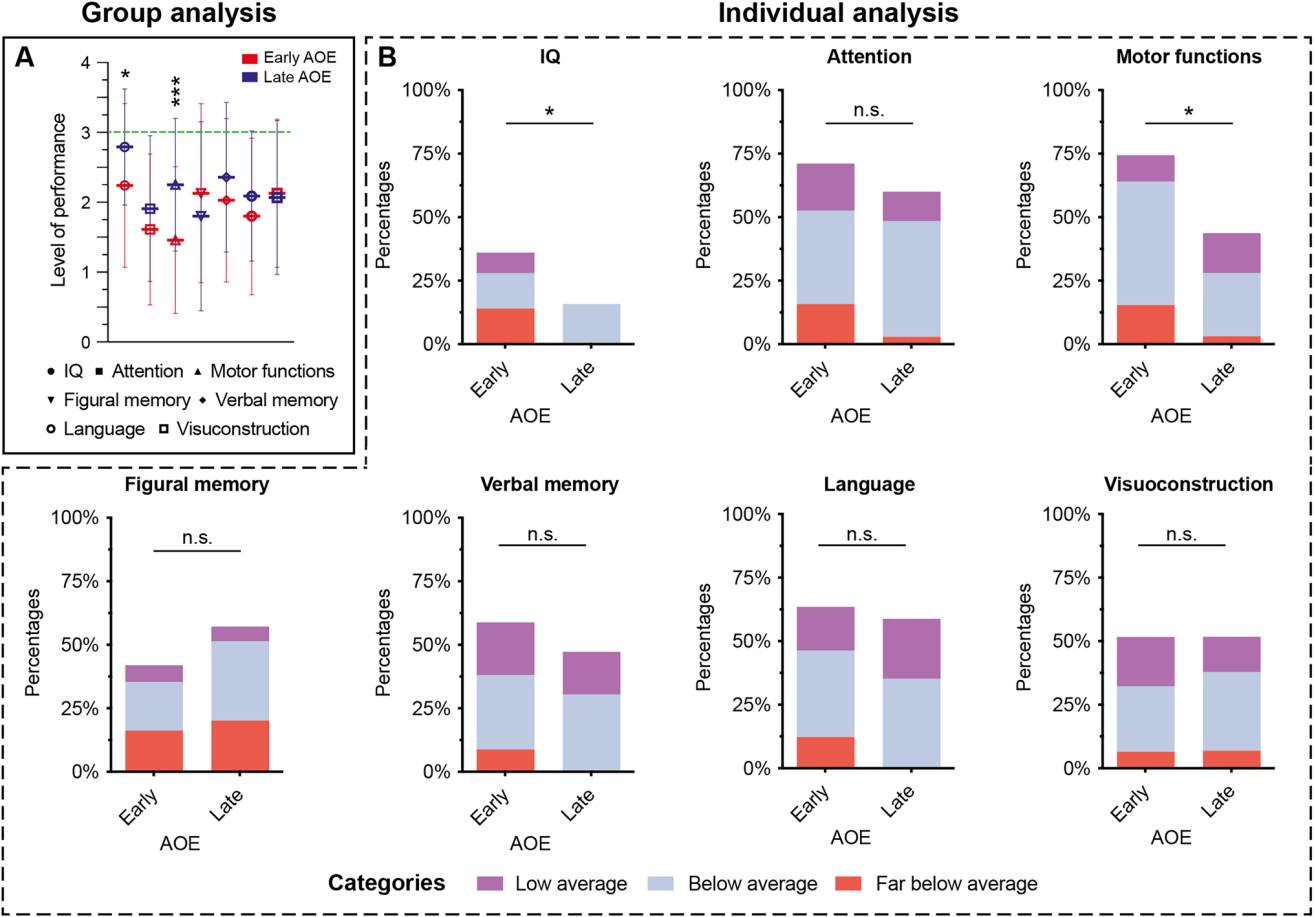
Cognitive performance across cognitive domains in patients with early versus late AOE. **A** Group analysis results: Average cognitive performance scores for patients with early and late AOE, presented as mean ± SD. The green line indicated the performance level ‘average’. **B** Individual analysis results: Proportion of impaired patients categorized as borderline, below average, and far below average based on their performance in neurocognitive assessments. *AOE* age at onset of epilepsy, *IQ* intelligence quotient, *n.s.* not significant, *SD* standard deviation; * *p* < 0.05, ***, *p* < 0.001

Across the entire study cohort, patients showed the lowest average performance in the cognitive domains of attention, motor functions, and language, with mean scores below the low average level (Tables 2 and 3; Figs. 2 and 3). The mean scores of all seven cognitive domains were below performance level “average”. While the number of missing data points varied across cognitive domains, each domain retained a sufficiently representative dataset for analysis.

**Table 2:** Analyses of Cognitive Performances for FCD II types

Cognitive domain		FCD IIa *n* = 19–22	FCD IIb *n* = 34–46	Total	Test statistics
IQ (*n* = 77)	M/SD n/% ↓	2.58/1.01 6/25.0%	2.49/1.09 14/26.5%	2.52/1.06 20/26.0%	*F*(1) = 0.03, *p* = 0.86 *χ*^2^(1) = 0.02, p = 0.90
Attention (*n* = 65)	M/SD n/% ↓	1.91/1.14 12/57.1%	1.71/1.05 30/68.2%	1.77/1.07 42/64.6%	*F*(1,62) = 0.59, *p* = 0.44 *χ*^2^(1) = 0.76, p = 0.38
Motor functions (*n* = 64)	M/SD n/% ↓	2.21/0.85 10/52.6%	1.71/1.14 27/60.0%	1.86/1.08 37/57.8%	*F*(1,61) = 2.52, *p* = 0.12 *χ*^2^(1) = 0.30, p = 0.59
Figural memory (*n* = 59)	M/SD n/% ↓	2.37/1.26 8/42.1%	1.83/1.30 20/50.0%	2.00/1.30 28/47.5%	*F*(1,56) = 1.29, *p* = 0.26 *χ*^2^(1) = 0.32, p = 0.57
Verbal memory (*n* = 63)	M/SD n/% ↓	2.25/1.07 11/55.0%	2.19/1.18 22/51.2%	2.21/1.14 33/52.4%	*F*(1,60) = 0.03, *p* = 0.88 *χ*^2^(1) = 0.08, p = 0.78
Language (*n* = 68)	M/SD n/% ↓	2.29/0.96 10/47.6%	1.83/1.11 29/61.7%	1.97/1.08 39/57.4%	*F*(1,65) = 2.72, *p* = 0.10 *χ*^2^(1) = 1.18, p = 0.28
Visuo-construction (*n* = 53)	M/SD n/% ↓	2.37/1.07 7/36.8%	2.00/0.98 20/58.8%	2.13/1.02 27/50.9%	*F*(1,50) = 1.50, *p* = 0.23 *χ*^2^(1) = 2.36, p = 0.13

Frequency distributions were analyzed using χ2-tests

*FCD* focal cortical dysplasia, *IQ* intelligence quotient, *M* mean, *n* number, *SD* standard deviations, ↓ impaired patients

**Table 3:** Analyses of Cognitive Performances for Early versus Late AOE

Cognitive domain		Early AOE *n* = 31–50	Late AOE *n* = 29–39	Total	Test statistics
IQ (*n* = 88)	M/SD n/% ↓	2.24/1.17 18/36%	2.79/0.83 6/15.8%	2.48/1.07 24/27.3%	*F*(1,85) = 5.55, *p* = 0.021 *χ*^2^(1) = 4.45, p = 0.035
Attention (*n* = 73)	M/SD n/% ↓	1.61/1.08 27/71.1%	1.91/1.04 21/60.0%	1.75/1.06 48/65.8%	*F*(1,70) = 1.57, *p* = 0.22 *χ*^2^(1) = 0.99, p = 0.32
Motor functions (*n* = 71)	M/SD n/% ↓	1.46/1.05 29/74.4%	2.25/0.95 14/43.8%	1.82/1.07 43/60.6%	*F*(1,68) = 10.45, *p* = 0.002 *χ*^2^(1) = 6.90, p = 0.009
Figural memory (*n* = 66)	M/SD n/% ↓	2.13/1.28 13/41.9%	1.80/1.35 20/57.1%	1.95/1.32 33/50.0%	*F*(1,63) = 1.18, *p* = 0.28 *χ*^2^(1) = 1.52, p = 0.22
Verbal memory (*n* = 70)	M/SD n/% ↓	2.03/1.17 20/58.8%	2.36/1.07 17/47.2%	2.20/1.12 37/52.9%	*F*(1,67) = 1.44, *p* = 0.24 *χ*^2^(1) = 0.94, p = 0.33
Language (*n* = 75)	M/SD n/% ↓	1.80/1.12 26/63.4%	2.09/0.93 20/58.8%	1.93/1.04 46/61.3%	*F*(1,72) = 1.35, *p* = 0.25 *χ*^2^(1) = 0.17, p = 0.68
Visuo-construction (*n* = 60)	M/SD n/% ↓	2.13/1.06 16/51.6%	2.07/1.10 15/51.7%	2.10/1.07 31/51.7%	*F*(1,57) = 0.06, *p* = 0.80 *χ*^2^(1) = 0.00, p = 0.99

Frequency distributions were analyzed using χ2-tests

*IQ* intelligence quotient, *M* mean, n number, SD standard deviations, ↓ impaired patients

### Comparative analysis of FCD types IIa and IIb

When comparing the FCD types (type IIa: *n* = 26, type IIb: *n* = 59, sample size: *n* = 85), gender (*p* = 0.86), as well as localization (*p* = 0.21) were found to be equally distributed (Supplementary Table S3). In addition, other clinical features such as the number of seizures per month (*p* = 0.74) or the ASM drug load (*p* = 0.1) did not significantly differ between the two groups. The duration of epilepsy until cognitive testing was longer in patients with FCD type IIb (18.8 ± 13.61 vs. 11.88 ± 9.09 years, *p* = 0.008) whereas the age at presurgical neurocognitive testing was not significantly different (20.35 ± 11.84 vs. 25.10 ± 15.64 years, *p* = 0.13). FCDs located in the left hemisphere were more common to be a FCD type IIa than IIb (69.2% vs. 42.2%, *p* = 0.022). Patients with FCD type IIa more often had a late AOE (61.5% vs. 34.5%, *p* = 0.021).

At group level, there were no significant differences in the mean cognitive performance scores across all domains in the presurgical neurocognitive testing for FCD II types IIa and IIb (Fig. 2A). However, significant differences were noted at the individual level (Fig. 2B). Specifically, in the cognitive domains of motor functions and figural memory, the"low average"category was significantly more common in type IIa than in IIb. This finding is somewhat mitigated by the fact that the overall proportion of cognitively impaired patients did not significantly differ between the two groups, with 25% versus 26.5% for motor functions and 42.1% versus 50% for figural memory (motor functions: χ^2^(1) = 0.30, *p* = 0.59; figural memory: χ^2^(1) = 0.32, *p* = 0.57, see Table 2).

### Influence of age at onset of epilepsy on cognitive performance

When comparing the early versus late AOE groups, gender (p = 0.54), duration of epilepsy (*p* = 0.64), ASM drug load (*p* = 0.09), hemisphere (p = 0.54) as well as localization (*p* = 0.84) were equally distributed (Supplementary Table S4). However, a notable difference was observed in the proportion of FCD type IIa and IIb between the groups whereby in the group of patients with early AOE the FCD type IIb was more often (early AOE in patients with IIa vs. IIb: 38.5% vs. 65.5%, *p* = 0.02).

At group level, significant main effects of AOE were found for IQ (F(1,85) = 5.55, *p* = 0.021) and motor functions (F(1,68) = 10.45, *p* = 0.002) (Table 3; Fig. 3A). Patients with early AOE exhibited significantly lower mean values in IQ (2.24 ± 1.17) and motor functions (1.46 ± 1.05) as compared to patients with late AOE (IQ: 2.79 ± 0.83; motor functions: 2.25 ± 0.95). The number of preoperative ASMs, entered as a covariate in the model, reached statistical significance only for figural memory in relation to AOE (F(1,63) = 5.01, *p* = 0.029), and not in the two cognitive domains that showed significantly mean differences.

At the individual level, the proportion of cognitively impaired patients in each cognitive domain was assessed and compared between patients with early and late AOE. Consistent with the group analysis, significant differences were found in the proportions of impaired patients for the cognitive domains IQ (χ^2^(1) = 4.45, *p* = 0.035) and motor functions (χ^2^(1) = 6.90, *p* = 0.009) between the early and late AOE groups (Table 3; Fig. 3B). Patients with early AOE were significantly more likely to be impaired (IQ: 36.0%; motor functions: 74.4%) compared to patients with late AOE (IQ: 15.8%; motor functions: 43.8%). Observed for IQ, patients with early AOE were more frequently categorized as “far below average” as compared to those with late AOE (14% vs. 0%, χ^2^(3) = 9.62, *p* = 0.022) (Fig. 3B). Similar differences were found in motor functions (χ^2^(3) = 9.86, *p* = 0.02), with early AOE patients more frequently categorized as “below average” as compared to late AOE patients (48.72% vs. 25%).

When comparing seizure frequency between patients with early and late AOE, those with early AOE showed a higher mean number of seizures per month (45 vs. 24; F(1) = 5.47, *p* = 0.022). However, when seizure frequency was categorized into three levels according to Vickrey et al. (as described in the Methods section), the distribution did not differ significantly between the early and late AOE groups (χ^2^(2) = 2.015, *p* = 0.365).

### Impact of other clinical variables on cognitive performance

No significant differences were detected dependent on lateralization (right vs. left hemisphere) or localization (frontal vs. temporal vs. posterior) of the epilepsy in both group and individual level analyses: There were no notable differences in the mean cognitive performance scores across all domains, nor in the proportions of patients exhibiting impairments in any cognitive domain, as detailed in Supplementary Tables S5–S6, Supplementary Figures S1 and S2.

### Determinants of cognitive performance

Taken together the findings suggests that from the potential mediator variables such as the type of FCD, AOE, lateralization and localization, the AOE was the crucial determinant of cognitive performance scores. Patients with an earlier onset (before 6 years) generally exhibited worse cognitive performances across various domains compared to those with a later onset. This was evident in globally lower scores, particularly in IQ and motor functions. The number of ASMs preoperatively did not have a statistically significant effect on these two cognitive domains.

## Discussion

The present study examined the presurgical cognitive profiles across seven domains of nearly 100 patients with FCD and pharmaco-resistant epilepsy. Patients were categorized according to histopathological types, and additional clinical features such as AOE, lateralization and localization, ASM drug load, and seizure frequency as potential mediator variables.

Regarding patient characteristics, the FCD type IIb was the most common. Individuals with FCD type IIb were more likely to experience early seizure onset, had longer durations of epilepsy, and were more frequently diagnosed with lesions in the right hemisphere compared to those with type IIa. These findings align with those of Yao et al. [28], who reported an earlier AOE for FCD type IIb compared to IIa, and Krsek et al. [29], who found a marginally earlier AOE for type IIb, although it was not statistically significant. Contrary to our results, Kwon et al. [30] observed higher rates of early AOE in patients with FCD type IIa; however, it is important to note that they set the threshold for early AOE at 2 years. Interestingly, the mean AOE in this study was slightly higher than that reported in most other studies, such as those by Veersema et al. [12] and Kimura et al. [8]. However, some studies have reported even higher average AOE, reaching up to 11 years. Our findings support the previous research suggesting that FCD IIb is the most prevalent FCD type [1]. The overall IQ of our sample was higher than those of other FCD cohorts. These differences are likely influenced by clinical factors such as AOE, which was consistently lower in these other studies and associated with poorer cognitive outcomes (see further below in the Discussion).

Among all clinical factors that were compared, the most notable differences were observed between early and late AOE, particularly in the cognitive domains of IQ and motor functions. In these cognitive domains, patients with early AOE consistently scored lower on average than the late AOE group. The mean IQ scores in the early AOE group were just above low average, whereas the late AOE group’s average IQ was almost undeteriorated. Individual analyses reinforced these findings, showing more frequent and severe cognitive impairments in the early AOE group. Regarding motor functions, both groups scored below average, but impairment was more prevalent and severe in the early AOE group compared to the late AOE group. Between the histopathological types IIa and IIb, FCD IIb generally scored lower across all cognitive domains, although differences in mean preoperative cognitive performance were not statistically significant. However, there were significant discrepancies in the proportions of patients with impairments in motor functions and figural memory. More patients with FCD type IIa fell into the low average category, while those with type IIb exhibited more severe impairments and a higher overall proportion of impaired patients. Other potential mediator variables, such as lateralization and localization, did not show any significant differences in mean cognitive performance scores or in the proportion of impaired patients in both group and individual analyses. Thus, among the factors analyzed, preoperative cognitive profiles in patients with FCD II seems to be predominantly influenced by AOE. As IQ is a comprehensive measure of general cognitive capabilities, the early AOE group exhibited significantly lower general cognitive capabilities compared to the late AOE group. The pronounced differences in motor functions further highlight specific deficits in this domain.

The limited number of studies that investigated preoperative cognitive profiles specifically for FCD types did not identify any notable differences [9, 16]. Consistent with this, the current study identified only minimal differences in cognitive profiles between the FCD types. Clinically, patients with FCD type IIb experienced longer epilepsy durations at the time of testing, although seizure frequency and ASM drug load were similarly distributed between the groups, suggesting no apparent clinical severity difference [31]. Furthermore, differences in the AOE were observed, with FCD type IIb patients more frequently having an early AOE. The observed earlier AOE in FCD type IIb compared to type IIa could be interpreted from a neurodevelopmental perspective. Previous studies have proposed that cellular markers associated with FCD type IIb emerge earlier during embryonic development [32]. Thus, type IIa would allow neuronal networks to develop more maturely than in case of type IIb, where epilepsy onset might occur earlier in the developmental timeline. Additional evidence supporting this notion comes from studies identifying balloon cells, a characteristic feature of FCD type IIb, as being of glial origin [33]. Glial cells appear early during corticogenesis, and their malformation would subsequently disrupt brain development at an earlier stage.

The data support the assumption that the AOE has a more significant impact on cognitive outcomes than the histopathological classification. Several studies have demonstrated that an earlier AOE is associated with lower IQ levels in patients with epilepsy across various underlying pathologies, with most research focusing on temporal lobe epilepsy (TLE) [12, 18, 34, 35]. Furthermore, a study by Cormack et al. highlighted that an earlier AOE was a robust predictor of intellectual impairment and that patients with an AOE after the age of 5 years were at a significantly reduced risk of intellectual dysfunction in pediatric TLE [36]. Only a few studies have linked a low IQ with an earlier AOE in patients with FCD [8, 12]. Unfortunately, these studies only used IQ as an indicator of cognitive impairment without specific testing for other cognitive domains. To our knowledge, there are no studies on the specific patient cohort of individuals with FCD and pharmacoresistant epilepsy that link cognitive deficits in various cognitive domains to the AOE, particularly motor deficits.

In the group of patients with early AOE, the average frequency of seizures was higher than in the late AOE group. Importantly, the ASM drug load was the same across both groups and did not influence cognitive performances when accounted for as a covariate in the analysis. To the best of our knowledge, no study has clearly demonstrated an association between increased seizure frequency and early AOE, but Kimura et al. identified a higher seizure frequency pattern as exacerbating factors for development quotient or intelligence quotient (DQ-IQ) decline in a multiple regression analysis in patients with FCD [8]. Therefore, the elevated seizure frequency might reflect more significant structural damage in early brain development, which consequently leads to poorer cognitive development.

Although our study incorporated a wide range of clinical variables to identify factors that could explain cognitive differences in patients with FCD, it lacked molecular data on the resected brain tissue. Honke et al. have tried to evaluate the relationship of genotype and phenotype patients with FCDs type II and intractable epilepsy [37]. Nearly half of the patients exhibited mutations in the GATOR1 complex encoding genes, such as *DEPDC5* and *NPRL3*, or gain-of-function variants in *mTOR*, with GATOR1 variants appearing exclusively in FCD IIa and *mTOR* variants present in both variants. These mutations corresponded with unique phenotypes, MRI patterns, and varying outcomes. However, conclusions are limited as only 17 patients were tested. Unfortunately, only few clinical data are shown and the data on cognitive performance of these patients are missing.

To better understand cognitive trajectories in FCD, it is essential to explore the relationships between AOE, histopathology, molecular data, and cognitive performance. Incorporating molecular data could reveal genetic drivers of FCD, aiding in predicting neurocognitive outcomes and planning early interventions. In addition, identifying mutations may guide pharmacological treatments and support the development of targeted therapies.

## Limitations

The study has several limitations and weaknesses that should be acknowledged. Conducted retrospectively at a single center, the design limits generalizability and the ability to infer causality. Prospective, longitudinal and multicentric studies are needed for delineating causality and monitoring cognitive performance trajectories over time. The analysis of categorized domains instead of test parameters of individual tests was necessary because not all patients could be assessed with the same tools. This may level fine graded differences which might have been seen with homogeneous tests. Furthermore, not all cognitive domains were assessed in every patient; in particular, several data points were missing in the domains of figural memory and visuo-construction. In addition, variables that could significantly impact findings, such as individual histopathological and molecular data, as well as quantitative MRI data, were not included in the analysis.

## Conclusions

The study highlights the substantial impact of early AOE on presurgical cognitive performance in patients with FCD. Patients with early AOE showed lower cognitive scores and a higher prevalence of cognitive impairment compared to those with late AOE. While pharmacoresistant epilepsy is more prevalent among patients with FCD type IIb, the FCD type did not significantly affect cognitive scores and only slight differences could be seen. Other potential impact factors, such as lateralization and localization, did not show any significant differences in mean cognitive performance scores on a group level or in the proportion of impaired patients on an individual level. Thus, AOE seems to be the most critical factor in determining preoperative cognitive capabilities in patients with FCD and intractable epilepsy. As expected in cases of early childhood onset epilepsies, a neurodevelopmental disruption, particularly in IQ and motor function, was seen in patients with early AOE. Future studies should take molecular characteristics into consideration.

## Supplementary Information

Below is the link to the electronic supplementary material.

Supplementary file1 (DOCX 430 KB)
