# Supplementary material for: Age at onset of epilepsy shapes neurocognitive profiles in focal cortical dysplasia
Source: J Neurol. 2025 May 3;272(5):373. doi: 10.1007/s00415-025-13090-4 (PMC12049294; doi:10.1007/s00415-025-13090-4)
Supplement: Supplementary file 1 — Supplementary file1 (DOCX 430 KB) [file 415_2025_13090_MOESM1_ESM.docx]

**Age at onset of epilepsy shapes neurocognitive profiles in focal cortical dysplasia**

Anna-Laura Potthoff^1^, Lukas Tennie^2^, Juri-Alexander Witt^2^, Attila Rácz^2^, Valeri Borger^1^, Hartmut Vatter^1^, Albert Becker^3^_,_ Rainer Surges^2^, Matthias Schneider^1^**^*^**, Christoph Helmstaedter^2^ **^*^**

*^1^Department of Neurosurgery, University Hospital Bonn, Venusberg Campus 1, 53127 Bonn, Germany*

*^2^Department of Epileptology, University Hospital Bonn, Venusberg Campus 1, 53127 Bonn, Germany*

*^3^Department of Neuropathology, University Hospital Bonn, Venusberg Campus 1, 53127 Bonn, Germany*

* Contributed equally

**Supplementary Figure S1**

Proportions of impaired patients per cognitive domain


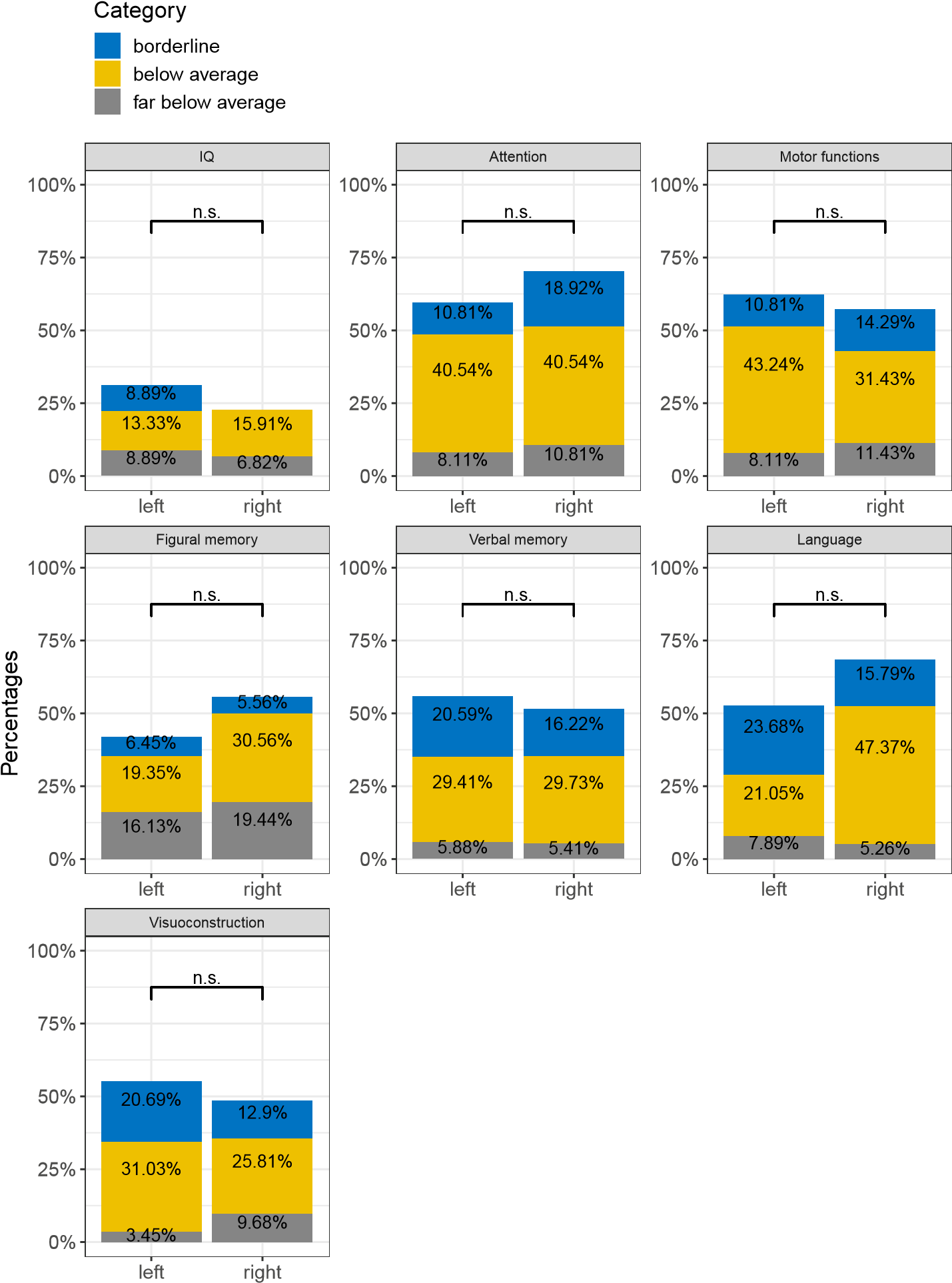


**Supplementary Figure S1.** Cognitive Performance across Cognitive Domains in Patients with FCD in Right versus Left Hemisphere.

Individual analysis results: Proportion of impaired patients categorized as borderline, below average, and far below average based on their performance in neurocognitive assessments. Abbreviations: n.s., not significant

**Supplementary Figure S2**

Proportions of impaired patients per cognitive domain


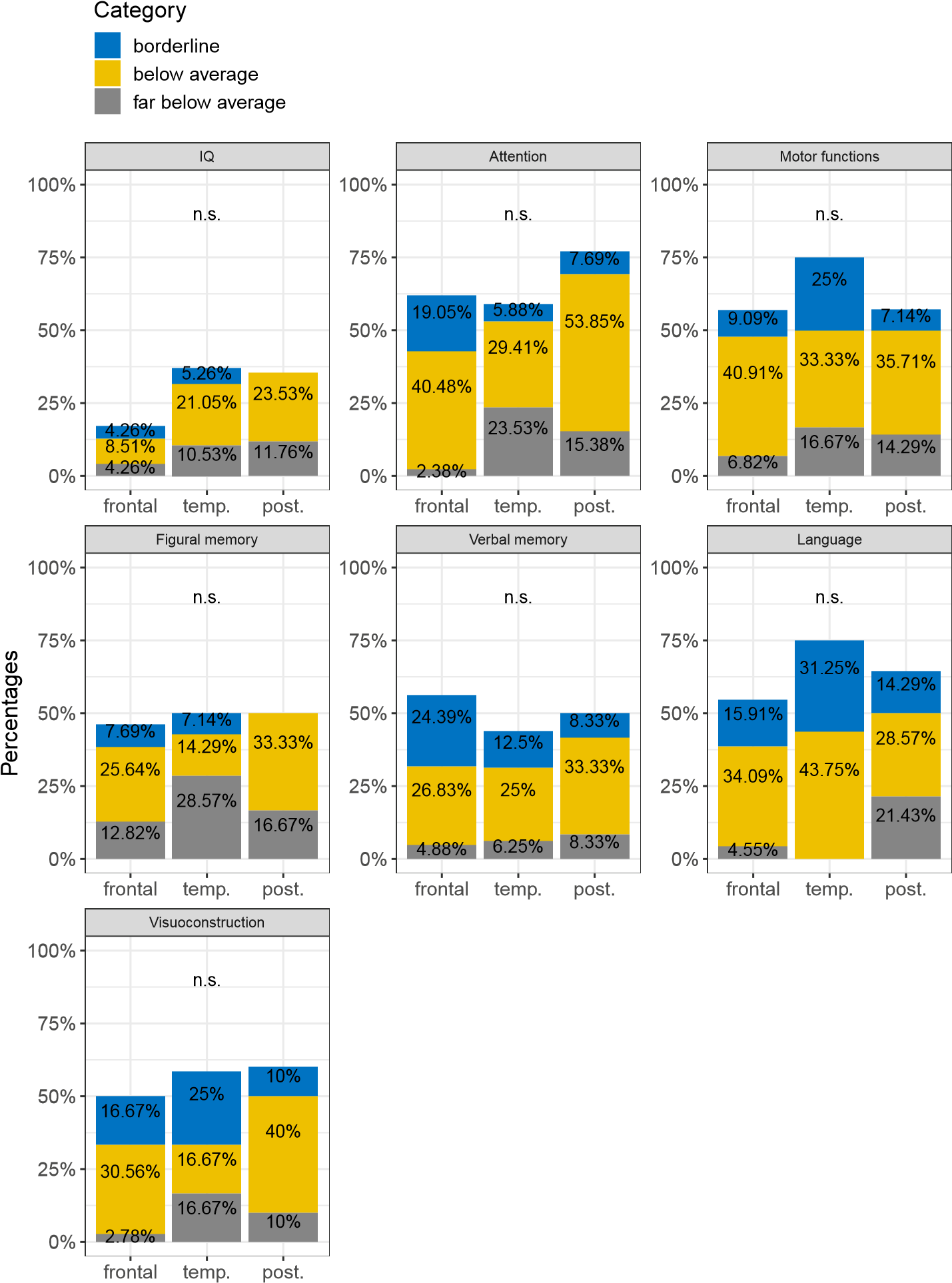


**Supplementary Figure S2.** Cognitive Performance Across Cognitive Domains in Patients with FCD in Frontal, Temporal, versus Posterior Localization.

Individual analysis results: Proportion of impaired patients categorized as borderline, below average, and far below average based on their performance in neurocognitive assessments. Abbreviations: n.s., not significant; temp, temporal; post, posterior

**Supplementary Table S1.** Patient Demographics and Clinical Characteristics

|  | **All patients**  n = 98 |
| --- | --- |
| Gender  Female  Male | 46 (47%)  52 (53%) |
| Age at seizure onset (yrs)* | 7.32 (7.48) |
| Age at surgery (yrs)* | 24.92 (14.79) |
| Duration of epilepsy (yrs)* | 17.44 (12.98) |
| Handedness  Right  Left  Ambidextrous | 80 (82%)  14 (14%)  4 (4%) |
| Mean IQ | 92.43 (14.79) |
| Seizures per month* | 37.51 (42.37) |
| ASM  Polytherapy  2 ASMs  3 ASMs  4 ASMs  Monotherapy  No ASM | 79 (81%)  43 (44%)  29 (30%)  7 (7%)  17 (17%)  2 (2%) |
| Lateralization  Right  Left | 48 (49%)  50 (51%) |
| Localization  Frontal  Temporal  Posterior  Others | 53 (54%)  21 (22%)  17 (17%)  7 (7%) |
| FCD Type  Ia  IIa  IIb  IIIa  Undefined Type | 2 (2%)  26 (27%)  59 (60%)  1 (1%)  10 (10%) |

*Values presented as mean (SD)

Abbreviations: ASM, antiseizure medication; FCD, focal cortical dysplasia; IQ, intelligence quotient; yrs, years

**Supplementary Table S1.** Neuropsychological Test Used for Patients’ Assessment

| **Domain** | **Test** | **Reference** |
| --- | --- | --- |
| Intelligence  Adults | Hamburg Wechsler  Intelligence Scales for Adults - revised  (HAWIE-R) | Tewes (1991) |
|  | Mehrfachwahl-Wortschatz-Intelligenztest  (MWT-B) | Lehrl (2005) |
|  | Standard Progressive Matrices (SPM) | Raven and Horn (2009) |
| Children | Vineland Adaptive Behavior  Scales (VABS) | Sparrow (2011) |
|  | K-ABC: Kaufman  Assessment Battery for  Children | Rollett and Preckel (2012) |
|  | Wechsler Intelligence Scale for Children, third edition | Tewes (2002) |
|  | Coloured Progressive  Matrices (CPM) | Raven et al. (2006) |
| Motor functions | Luria Motor Sequences | Luria and Critchley (1970) |
|  | Purdue Pegboard Test (PPT)  Finger Tapping Test (FTT) | Ventola (2013)  Schmitt (2013) |
| Attention/executive fct. | d2-Letter cancellation Aufmerksamkeits-Belastungs-Test | Brickenkamp (2002) |
|  | Trail-Making Test  c.I.-Test | Reitan (1992)  Lehrl (1984) |
|  | Digit-span Task backward  Corsi-block-tapping Test backward | Tewes (1991)  Corsi (1973) |
|  | Maze Test (Chapuis) | Chapuis (1959) |
| Memory  Verbal memory | Digit-span Task forward  Verbaler Lern- und  Merkfähigkeitstest (VLMT) | Tewes (1991  Helmstaedter (2001) |
| Figural memory | Corsi-block-tapping Test forward  Diagnosticum für  Cerebralschädigung - revised (DCS-R) | Corsi (1973)  Helmstaedter (1991) |
| Language | Phonematic Fluency (LPS)  Token Test  Boston Naming Test | Horn (1983)  De Renzi (1962)  Busch (2005), Kaplan (2001) |
| Visuoconstruction | Block Design Test | Tewes (1991) |
|  | Mental Rotation Task LPS-7 | Horn (1983) |

| **Patients’ characteristics** | **Sample (n=85)** | **FCD IIa (n=26)** | **FCD IIb (n=59)** | **Statistics** |  |
| --- | --- | --- | --- | --- | --- |
| Gender  Male | 47 (55.3%) | 14 (53.8%) | 33 (55.9%) | *χ*^2^(1) = 0.32, |  |
| Female | 38 (44.7%) | 12 (46.2%) | 26 (44.1%) | *p* = 0.86 |  |
| Age of onset  Mean (SD) | 6.79 (6.92) | 8.46 (8.53) | 6.03 (6.00) | *F*(1,83) = 2.24 |  |
| Range | 0-43 | 1-43 | 0-41 | *p* = 0.14 |  |
| Early vs. late AOE  Early | 48 (57.1%) | 10 (38.5%) | 38 (65.5%) | *χ*^2^(1) = 5.37, |  |
| Late | 36 (42.9%) | 16 (61.5%) | 20 (34.5%) | *p* = 0.021 |  |
| Age at surgery  Mean | | 23.65 (14.68) | 20.35 (11.84) | 25.10 (15.64) | *F*(1) = 2.37, |
| Range | | 3-70 | 4-56 | 3-70 | *p* = 0.13 ^†^ |
| Duration of epilepsy  Mean | 16.68 (12.76) | 11.88 (9.09) | 18.80 (13.61) | *F*(1) = 7.56, |  |
| Range | 0-63 | 0-36 | 0-63 | *p* = 0.008 ^†^ |  |
| Lobe | n = 82 | n = 26 | n = 56 |  |  |
| Frontal | 49 (59.8%) | 13 (50.0%) | 36 (64.3%) | *χ*^2^(2) = 3.12, |  |
| Temporal | 16 (19.5%) | 8 (30.8%) | 8 (13.3%) | *p* = 0.21 |  |
| Posterior | 17 (20.7%) | 5 (19.2%) | 12 (21.4%) |  |  |
| Hemisphere  Right | 42 (49.4%) | 8 (30.8%) | 34 (57.6%) | *χ*^2^ (1) = 5.21, |  |
| Left | 43 (50.6%) | 18 (69.2%) | 25 (42.2%) | *p* = 0.022 |  |
| Seizures (per month)  Mean (SD) | 38.35 (42.22) | 35.64 (46.22) | 39.58 (40.82) | *F*(1) = 0.11, |  |
| Range | 0.16-4500 | 0.16-4500 | 0.25-300 | *p* = 0.74 |  |
| Number of ASMs  Mean (SD) | 2.26 (0.89) | 2.50 (0.81) | 2.15 (0.91) | *F*(1) = 2.82 |  |
| Range | 0-4 | 1-4 | 0-4 | *p* = 0.10 |  |

**Supplementary Table S3.** Comparison of Patients’ Demographics and Characteristics Between FCD Type IIa and IIb

Frequency distributions were analyzed using χ2-tests.

Abbreviations: ASM, antiseizure medication; FCD, focal cortical dysplasia; SD, standard deviations

**Supplementary Table S4.** Comparison of Patients’ Demographics and Characteristics Between Early and Late AOE

| **Patients’ characteristics** | **Total (n=97)** | **Early AOE**  **(n=52)** | **Late AOE (n=45)** | **Statistics** |
| --- | --- | --- | --- | --- |
| Gender  Male | 51 (52.6%) | 29 (56.9%) | 23 (50.0%) | *χ*^2^(1) = 0.46, |
| Female | 46 (47.4%) | 22 (43.1%) | 22 (50.0%) | *p* = 0.50 |
| Duration of Epilepsy  Mean | 17.47 (13.04) | 16.73 (12.53) | 18.22 (13.69) | *F*(1) = 0.36, |
| Range | 0-63 | 0-52 | 0-63 | *p* = 0.55 |
| Hemisphere  Right | 47 (48.5%) | 23 (48.9%) | 29 (58.0%) | *χ*^2^ (1) = 0.80, |
| Left | 50 (51.5%) | 24 (51.1%) | 21 (42.0%) | *p* = 0.37 |
| Lobe | n =91 | n = 48 | n = 43 |  |
| Frontal | 53 (58.2%) | 28 (58.3%) | 25 (58.1%) | *χ*^2^(2) = 0.47, |
| Temporal | 21 (23.1%) | 10 (20.8%) | 11 (25.6%) | *p* = 0.79 |
| Posterior | 17 (18.7%) | 10 (20.8%) | 7 (16.3%) |  |
| Seizures (per month)  Mean (SD) | 35.96 (39.10) | 45.13 (44.34) | 24.78 (28.43) | *F*(1) = 5.47, |
| Range | 0.16 -150 | 0.25-150 | 0.16-100 | *p* = 0.022 ^†^ |
| Number of ASMs  Mean (SD) | 2.22 (0.89) | 2.15 (1.0) | 2.29 (0.76) | *F*(1) = 0.55, |
| Range | 0-4 | 0-4 | 1-4 | *p* = 0.46 |

Frequency distributions were analyzed using χ2-tests.

Abbreviations: ASM, antiseizure medication; AOE, age at onset of epilepsy; SD, standard deviations

| **Test category** |  | **Right hemisphere**  (n = 31-43) | **Left hemisphere**  (n=29-45) | **Total** | **Test statistics** |
| --- | --- | --- | --- | --- | --- |
| IQ (n = 89) | M/SD n/% ↓ | 2.54/1.07 10/22.7% | 2.42/1.08 14/31.1% | 2.48/1.07 24/27.0% | *F*(1,86) = 0.25, *p* = .62 *χ*^2^(1) = 0.79, p = .37 |
| Attention (n = 74) | M/SD n/% ↓ | 1.68/1.03 26/70.3% | 1.86/1.11 22/59.5% | 1.77/1.07 48/64.9% | *F*(1,71) = 0.57, *p* = .45 *χ*^2^(1) = 0.95, p = .33 |
| Motor functions (n = 72) | M/SD n/% ↓ | 1.89/1.11 20/57.1% | 1.78/1.06 23/62.2% | 1.83/1.07 43/59.7% | *F*(1,69) = 0.14, *p* = .71 *χ*^2^(1) = 0.19, p = .66 |
| Figural memory (n = 67) | M/SD n/% ↓ | 1.81/1.31 20/55.6% | 2.16/1.32 13/41.9% | 1.97/1.31 33/49.3% | *F*(1,64) = 1.13, *p* = .29 *χ*^2^(1) = 1.24, p = .27 |
| Verbal memory (n = 71) | M/SD n/% ↓ | 2.24/1.21 19/51.4% | 2.09/1.08 19/55.9% | 2.17/1.15 38/53.5% | *F*(1,68) = 0.31, *p* = .58 *χ*^2^(1) = 0.15, p = .70 |
| Language (n = 76) | M/SD n/% ↓ | 1.76/1.03 26/68.4% | 2.13/1.04 20/52.6% | 1.95/1.04 46/60.5% | *F*(1,73) = 2.40, *p* = .13 *χ*^2^(1) = 1.98, p = .16 |
| Visuo-  construction (n = 60) | M/SD n/% ↓ | 2.10/1.14 15/48.4% | 2.10/1.01 16/55.2% | 2.10/1.07 31/51.7% | *F*(1,57) = 0.00, *p* = .98 *χ*^2^(1) = 0.28, p = .60 |

**Supplementary Table S5.** Analyses of Cognitive Performances in Right versus Left Hemisphere

Frequency distributions were analyzed using χ2-tests.

Abbreviations: IQ, intelligence quotient; M, mean; n, number; SD, standard deviations; ↓, impaired patients

| **Test category** |  | **Frontal**  (n = 45-54) | **Temporal**  (n = 17-19) | **Posterior**  (n = 13-17) | **Total** | **Test statistics** |
| --- | --- | --- | --- | --- | --- | --- |
| IQ (n=83) | M/SD n/% ↓ | 2.72/0.87  8/17.0% | 2.26/1.19  7/36.8% | 2.23/1.25  6/35.3% | 2.52/1.05 21/25.3% | *F*(2,79) = 2.21, *p* = .12 *χ*^2^(2) = 3.94, p = .14 |
| Attention (n=72) | M/SD n/% ↓ | 1.95/0.99 26/61.9% | 1.65/1.27 10/58.8% | 1.38/1.04 10/76.9% | 1.78/1.08 46/63.9% | *F*(2,68) = 1.54, *p* = .22 *χ*^2^(2) = 1.22, p = .54 |
| Motor functions (n=70) | M/SD n/% ↓ | 1.89/1.06 25/56.8% | 1.58/1.08  9/75.0% | 1.79/1.19  8/57.1% | 1.81/1.08 42/60.0% | *F*(2,66) = 0.37, *p* = .69 *χ*^2^(2) = 1.36, p = .51 |
| Figural memory (n= 65) | M/SD n/% ↓ | 2.10/1.25 18/46.2% | 1.93/1.54  7/50.0% | 1.83/1.27  6/50.0% | 2.02/1.31 31/48.4% | *F*(2,61) = 0.33, *p* = .72 *χ*^2^(2) = 0.09, p = .96 |
| Verbal memory (n=69) | M/SD n/% ↓ | 2.12/1.03 23/56.1% | 2.44/1.32  7/43.8% | 2.17/1.34  6/50.0% | 2.20/1.15 36/52.2% | *F*(2,65) = 0.44, *p* = .64 *χ*^2^(2) = 0.73, p = .69 |
| Language (n=74) | M/SD n/% ↓ | 2.05/1.03 24/54.5% | 1.88/0.96 12/75.0% | 1.64/1.22  9/64.3% | 1.93/1.05 45/60.8% | *F*(2,70) = 0.82, *p* = .44 *χ*^2^(2) = 2.15, p = .34 |
| Visuo-  construction (n = 58) | M/SD n/% ↓ | 2.17/1.00 18/50.0% | 1.92/1.16  7/58.3% | 1.80/1.14  6/60.0% | 2.05/1.05 31/53.4% | *F*(2,54) = 0.59, *p* = .56 *χ*^2^(2) = 0.46, p = .80 |

**Supplementary Table S6.** Analyses of Cognitive Performances in Patients with FCD in Frontal, Temporal, versus Posterior Localization

Frequency distributions were analyzed using χ2-tests.

Abbreviations: IQ, intelligence quotient; M, mean; n, number; SD, standard deviations; ↓, impaired patients
